# External Lymphatic Fistula After Radical Surgery for Colorectal Cancer: A Case Series

**DOI:** 10.3390/cancers17091416

**Published:** 2025-04-23

**Authors:** Vincenzo Tondolo, Luca Emanuele Amodio, Federica Marzi, Giada Livadoti, Giuseppe Quero, Gianluca Rizzo

**Affiliations:** 1 Digestive and Colo-Rectal Surgery Unit, Ospedale Isola Tiberina Gemelli Isola, 00186 Rome, Italy; vincenzo.tondolo@fbf-isola.it (V.T.); lucaemanueleamodio@virgilio.it (L.E.A.); federicamarzi9@gmail.com (F.M.); giada.livadoti@fbf-isola.it (G.L.); 2 Dipartimento di Scienze Mediche e Chirurgiche, Università Cattolica del Sacro Cuore, 00168 Rome, Italy; giuseppe.quero@policlinicogemelli.it; 3 Digestive Surgery Unit, Fondazione Policlinico Universitario Agostino Gemelli IRCCS, 00168 Rome, Italy

**Keywords:** lymphatic fistula, chyloperitoneum, extensive lymphadenectomy, lymphatic leakage, colorectal surgery, total parenteral nutrition, conservative management

## Abstract

Background: The incidence of external lymphatic fistula (ELF) represents a relatively rare complication after surgery for colorectal cancer, especially in Western countries. However, the rate of this complication is progressively increasing following the introduction of complete mesocolic excision and central vascular ligation with consequent extensive lymphadenectomy. There are no guidelines for the management of ELF, with therapeutic options varying from conservative procedures to more invasive surgeries. The aim of this study was to retrospectively quantify the rate of ELF after surgery for colorectal cancer, to describe its management, and to evaluate its clinical impact on early postoperative outcomes in a tertiary referral European centre. Methods: Data on all patients who underwent surgery for colorectal cancer at our institution between July 2022 and December 2024 were entered into a database. Preoperative, perioperative, and early (within 30 days) postoperative data were recorded. Results: A total of 279 patients underwent elective surgery for colorectal cancer (205 colon and 74 rectum). No postoperative deaths occurred within 30 days after surgery, and the rates of overall and major (grade ≥ 3) postoperative morbidity were 34.7% and 7.1%, respectively. The anastomotic leakage and reoperation rates were 2.8% and 5.3%, respectively. ELFs occurred in 15 patients (5.3%). In all patients, conservative treatment (based on fasting, total parenteral nutrition (TPN), and a prolonged medium-chain triglyceride (MCT) diet) was administered successfully. A recurrent ELF (after the first oral feeding resumption) occurred in four (26.6%) patients, but all were successfully treated with a conservative approach. The occurrence of an ELF prolonged the postoperative length of stay which was 12 days, a length higher than that recorded in patients without ELF. Conclusions: The occurrence of an ELF was found to be a relatively frequent complication after surgery for colorectal cancer and appears to negatively influence only the postoperative length of stay. Conservative management appeared to be a successful treatment.

## Simple Summary

In this study, we evaluate the incidence of external lymphatic fistula (ELF) and its clinical impact on early postoperative outcomes among patients undergoing surgery for colorectal cancer. ELF occurred in 5.3% of patients, representing a relatively frequent complication after colorectal cancer surgery. However, conservative management, including fasting, total parenteral nutrition, and a prolonged medium-chain triglyceride diet, proved to be successful in all cases. In terms of post-operative outcomes, the occurrence of ELF was associated with a significant increase in postoperative hospital stay.

## 1. Introduction

External lymphatic fistula (ELF) is a post-surgical complication characterized by the output of a typical noninfected white milky fluid containing a high concentration of triglycerides from surgical drainage.

Damage to lymphatics and/or their ineffective closure during vessel dissection are recognized as the main pathogenic factors of ELF [1], leading to an occurrence rate between 1% and 11% after abdominal surgical procedures [2]. However, no guidelines are currently available in the literature for the clinical treatment of ELFs. A conservative approach, consisting of prolonged maintenance of abdominal drainage, together with diet modification (a fast and/or medium-chain triglyceride diet) and parenteral nutrition [1,3], is generally the most frequent and efficient treatment of choice. Conversely, a more invasive treatment, consisting of reoperation with surgical closure of the leakage also using fibrin glue, a peritoneal–venous shunt, and transabdominal catheterization of the cisterna chyli, is adopted in cases of persistent ELFs complicated by malnutrition and immune dysfunction [3].

In the context of colorectal surgery, current evidence indicates that the rate of ELF is slightly lower than that in other surgical procedures, with values ranging between 1% and 7.3% [4,5,6]. The pathogenesis of an ELF after colorectal surgery for cancer is not clear. For instance, extensive lymph node dissection during colorectal surgeries may cause iatrogenic injury to abundant lymphatics, such as the direct tributaries of the cisterna chyli (during left colectomy/anterior resection of the rectum) and the main lymphatic branches around the superior mesenteric artery and vein (during right/extended right colectomy), thereby increasing the occurrence of postoperative chylous ascites [7]. Moreover, the recent widespread use of the complete mesocolic excision (CME) technique and subsequent extensive (D3) lymph node dissection appear to have increased the risk of ELF, which in this context of patients ranges between 4.4% and 10%, as reported in more recent case series [6,8,9]. The relationship between extensive lymphadenectomy and a greater occurrence rate of ELF has been widely demonstrated in a series of East Asian countries, where extensive D3 lymphadenectomy is considered the standard surgical treatment for colorectal cancers [10]. Conversely, only a few reports, with a restricted number of patients, have been reported from Western countries [11], providing a limited overview of the incidence rate, consequences, and management strategies for ELFs in Europe.

Based on these premises, the aim of this study was to retrospectively quantify the rate of ELF after surgery for colorectal cancer, to describe its management, and to evaluate its clinical impact on early postoperative outcomes in a tertiary referral European centre.

## 2. Materials and Methods

### 2.1. Study Design and Population

All patients who underwent radical colorectal resection with an elective setting for histologically confirmed cancer from July 2022 to December 2024 at the Isola Tiberina Hospital, Gemelli Isola of Rome, were retrospectively enrolled in the study. All cases were preoperatively discussed by dedicated multidisciplinary tumour boards for colon and rectal diseases to assess the tumour staging and treatment strategy. Specifically, in cases of colon cancer, clinical staging and resectability were assessed based on preoperative whole-body computed tomography (CT) scans and indications following the most recent guidelines for colon cancer treatment [12]. In rectal cancer, tumour staging was completed by using pelvic magnetic resonance imaging (MRI) to evaluate the tumour location (intraperitoneal or extraperitoneal rectal cancer) and locoregional staging (early or locally advanced). The indications for upfront surgery or chemoradiotherapy were based on the most recent National Comprehensive Cancer Network (NCCN) guidelines [13]. Perioperative data were retrospectively collected from a prospectively maintained database. The clinical and demographic characteristics included age, sex, BMI, Charlson Comorbidity Index score, and American Society of Anaesthesiologists (ASA) physical status. The following perioperative features were also collected: tumour location, preoperative clinical stage, preoperative neoadjuvant chemotherapy and/or radiotherapy, type of surgical approach (laparoscopic or open), rate of conversion, intraoperative complications, days to resume oral intake after surgery, and short-term (within 30 days from surgery) postoperative complications (classified according to the Clavien–Dindo classification [14]). Among patients with rectal cancer, the site of the tumour was further classified as low rectum (from the internal anal orifice, i.a.o., to 5 cm from the i.a.o.), middle rectum (from 5 to 10 cm from the i.a.o.), or high rectum (from 10 to 15 cm from the i.a.o.). ELF was defined as a change in the output of abdominal drainage from serum to white milky fluid, with a high triglyceride concentration (triglyceride level of ≥110 mg/dL) and no signs of infection [15]. Moreover, ELF severity was graded according to the grading system proposed by van der Gaag et al. and based on the time of resolution: Grade A, time to resolution < 7 days; Grade B, time to resolution 7–14 days; and Grade C, time to resolution > 14 days or requiring surgical treatment [16]. Short-term postoperative mortality was also recorded and defined as any death occurring within 30 days after surgery. The following histopathological data were additionally evaluated: tumour size (maximum diameter in cm); number of harvested lymph nodes; number of metastatic nodes; and pT, pN, and TNM stages according to the AJCC 8th edition [17].

### 2.2. Operative Technique

#### 2.2.1. Right and Left Colon Cancer

Whenever feasible, a minimally invasive approach was adopted. All patients were subject to high-tie ligation of the main vessels with extensive lymph node dissection around the origin of the main vessels and complete mesocolic excision (CME) while preserving the integrity of the embryological planes. Formal right hemicolectomy was performed through extensive dissection of the superior mesenteric vein (SMV), thus clearing the duodenal C and the head of the pancreas from the right mesocolon tissues. Ligation of the ileocolic vessels and right colic (if present) was routinely performed. In the case of an extended right colectomy, the middle colic vessels were dissected at the origin. The left hemicolectomy was performed by tying the inferior mesenteric artery (IMA) 1 cm from the origin of the aorta and the inferior mesenteric vein (IMV) at the inferior margin of the pancreas. Oncologic resection of the splenic flexure included dissection of the lymph nodes at the origin of the IMA and resection of the left colic artery associated with the section of the left branches of the middle colic vessels. Independent of the type of procedure performed, the dissection phase was always performed using monopolar electrocautery (i.e., electrified crochets) or energy (ultrasound) devices. A drain was positioned in the abdomen near the anastomosis (in the case of formal/extended right colectomy) or in the pelvis (in the case of left colectomy) after surgery.

#### 2.2.2. Rectal Cancer

Anterior resection with partial mesorectal excision (PME) was the treatment of choice for upper rectal cancers. Conversely, patients with extraperitoneal rectal cancer underwent anterior resection with total mesorectal excision (TME) and colo-rectal or colo-anal anastomosis according to the technical feasibility. In the case of low rectal cancer, without oncologically free resection margins from the anal canal, abdominoperineal resection with TME was indicated. For left colectomy, the IMA and IMV were isolated and ligated at the origin and along the inferior border of the pancreas, respectively. Even in these cases, the dissection phase was always performed with monopolar or ultrasound energy devices [16]. A protective stoma was used for all patients at high risk (according to age, comorbidities, height of the colorectal anastomosis, and preoperative neoadjuvant chemoradiotherapy) for postoperative anastomotic leakage. A drain was always positioned near the anastomosis in the pelvis.

#### 2.2.3. Postoperative Management and ELF Treatment

An oral diet was progressively started on the first postoperative day. The abdominal drain was removed at the resumption of intestinal function (gas or stool canalization). Patients were discharged if no postoperative complications occurred during the fourth or fifth postoperative day. In the case of an ELF, fast and total parental nutrition (TPN) was immediately implemented and continued for at least 5 days. Neither somatostatin nor octreotide was administered to any patient. Oral refeeding with a mid-chain triglyceride (MCT) diet was then restarted after 5 days in the case of ELF resolution, which was defined as serous output from drainage. ELF was considered resolved if no white milky fluid from the drainage was detected for at least 48 h after the resumption of MCT. If the drainage output remained serous for almost 48 h from the start of refeeding, the drainage tube was removed, and the patient was discharged with a low-fat diet and a strictly ambulatory follow-up at 1 week and at 1 month from hospital discharge.

#### 2.2.4. Study Outcomes

The primary endpoint of this study was to retrospectively quantify the rate of ELF after surgery for colorectal cancer, describe its management, and evaluate its clinical impact on early postoperative outcomes. To evaluate the clinical impact of the occurrence of ELF on postoperative outcomes and to hypothesize potential predictive factors for the occurrence of ELF, we compared baseline and perioperative outcomes of patients who developed an ELF (ELF group) with patients who did not develop an ELF (No ELF group). However, due to the high discrepancy in sample size between the two groups, no statistical analysis about the significancy of the differences between two groups was performed.

## 3. Results

From July 2022 to December 2024, 279 patients (154 males, median age: 73 years, IQR 65–81 years) underwent elective surgery for colorectal cancer at our institution. The clinical–demographic characteristics and perioperative features of the study population are reported in Table 1. Table 2 shows the baseline characteristics and perioperative features of ELF and NO ELF groups. A total of 205 (73.5%) patients had colon cancer and 74 (26.5%) had rectal cancer, among which 50 (17.9%) had extraperitoneal (middle and low rectum) cancer. Neoadjuvant chemoradiation therapy was prescribed for 48 patients (17.2%). After 8 to 12 weeks, 41 patients underwent anterior resection with TME, and 9 underwent abdominoperineal resection with TME. A total of 257 (92.1%) surgical procedures were performed with a minimally invasive approach (233 with a laparoscopic approach and 24 with a robotic approach), and conversion to an open approach was necessary in 12 (4.3%) patients. About baseline characteristics, a high prevalence of right colon cancer was found in the ELF group (60% vs. 37.9% in the No ELF group) (Table 2).

At pathological evaluation of the specimens, the median number of harvested nodes was 22 (IQR: 16–30). Metastatic lymph nodes were also found in 99 (35.4%) patients. In the ELF group, we found a rate slightly higher than the No ELF group of pT4 cancers (26.7% vs. 17.0%), number of harvested lymph nodes (23 vs. 22), and patients with positive nodes (46.7% vs. 34.9%) (Table 2).

No postoperative deaths occurred within 30 days after surgery. A total of 97 (34.7%) patients experienced a complication within 30 days of surgery, but only 20 (7.1%) patients experienced complications with a grade ≥ 3 according to Clavien’s classification.

Overall, 15 patients (5.3%) developed postoperative ELF. Among these patients, nine (3.2%) had grade A ELF, and six (2.1%) had grade B ELF. ELF occurred after a median time of 3 days (range: 3–5 days) after surgery. A conservative approach based on fast and TPN was adopted for all patients, from whom serous non-chylous drainage fluid was obtained within 24 h. After 5 days of fasting and TPN, all patients started an MCT. After starting the MCT, four patients (26.6%) experienced a recurrence of chylous drainage within 24 h of the start of oral feeding. For these patients, a conservative approach based on fasting and TPN was adopted for an additional 5 days. After 5 days of fasting and TPN, an MCT diet was started, with no recurrences of ELF observed in any patients. Concomitant postoperative complications adjunctive to the ELF occurred in five patients: one patient with a pancreatic fistula after colonic resection of the splenic flexure with enlargement of the spleen and pancreatic tail, one patient with a gluteal abscess who underwent sigmoid colon cancer resection for metastatic sigmoid cancer, one patient with cholecystitis, one patient with pneumonia, and one patient with a fever of unknown origin. In all three cases, antibiotic therapy was required, leading to a complete resolution of the clinical condition. No postoperative deaths due to ELF within 30 days of surgery were recorded.

Overall, anastomotic leakage occurred in eight patients (2.8%). A surgical procedure to treat postoperative complications was needed for 15 (5.3%) patients. As reported in Table 2, patients who developed ELF did not develop a major postoperative complication (0%) or an anastomotic leak (0%) with no need of reoperation and no died within 30 days from surgery. The median overall postoperative length of stay was 5 days (IQR: 5–7 days). Among patients who developed an ELF, the median length of postoperative stay was 12 days (IQR: 10–14 days), higher than the median postoperative length of stay recorded in No ELF group (5 days, IQR: 5–6 days) (Table 2).

## 4. Discussion

The aim of the present study was to retrospectively quantify the rate of ELF after surgery for colorectal cancer in our institution. In our series, the occurrence of an ELF was reported at a rate of 5.3%. Postoperative ELF represents a relatively rare complication after abdominal surgery characterized by the output of noninfected, odourless, white, milky, triglyceride-rich (>110 mg/dL), and alkaline fluid from intra-abdominal drainage. The rate of incidence of ELF appeared to vary with the site of abdominal surgery. After pancreatic surgery, the rate ranged between 1.0% and 11% [18,19], and after gastrectomy, it ranged between 0.3 and 0.7% (after D2 lymphadenectomy) and 11.7% after D3 lymph node dissection [20,21]. After colorectal surgery, the incidence of ELF ranged between 1.0% and 6.6% [3,22,23]. The rate of ELF reported in our series is similar to that reported by Sun et al., who analyzed the risk of chylous ascites in 661 consecutive patients (over 6 years, 2012–2018) who underwent right-sided hemicolectomy with CME and D3 lymphadenectomy [6]. Moreover, after right hemicolectomy with CME, a greater rate of chylous ascites was observed, as reported in a study by Wang Y et al. In this study, 22 of 172 (12.8%) patients who underwent laparoscopic CME for right colon cancer (in 5 years, 2010–2015) experienced this complication [24]. In contrast, a recent multicentre study by Qin et al. involving 1090 right colon cancer patients who underwent laparoscopic D3 lymphadenectomy, showed that chylous ascites occurred in only 4.4% of patients [8]. The signs of ELF usually appear on the third or fourth day after primary surgery and are strictly related to the resumption of oral food intake, which is considered an important predisposing factor related to the occurrence of ELF [25]. The occurrence of ELF negatively influenced the postoperative recovery of patients, increasing the length of postoperative hospital stay and the risk of malnutrition, electrolyte imbalances, and immunosuppression due to lymphocyte loss.

Another aspect analyzed in our case series was the effect of the occurrence of an ELF on the postoperative outcome. In our experience, the occurrence of an ELF seems to negatively influence the length of postoperative hospital stay, which was 12 days, greater than the median length of postoperative stay recorded in no ELF group (5 days). The median length of hospital stay recorded among patients with ELF was generally within the range published by Qin et al., who reported a mean length of postoperative stay of approximately 11 days [8]. Matsuda et al. analyzed patients who underwent laparoscopic colorectal surgery and reported that the length of hospital stay among patients with ELF was significantly greater than that among patients without ELF (14 days vs. 10 days) [5]. The negative effect of the occurrence of ELF on postoperative length of stay represents the main and the only effect of ELF on postoperative outcomes.

Three main pathogenetic mechanisms have been described in the formation of an ELF or chyloperitoneum, including the presence of a lympho-peritoneal fistula associated with abnormal retroperitoneal lymphatic vessels, the exudation of chyle through the vessel wall without signs of a fistula, and exudation or leakage due to the rupture of dilated lymphatics in the bowel wall and mesentery due to vessel obstruction [26]. In our series, damage to the main tributaries of the cisterna chyli was plausible only for left-sided dissections (e.g., left hemicolectomy and rectal resection), which represent only 40% of cases. None of the described mechanisms were demonstrated in our series because no patient underwent reoperation. The most likely hypothesis is that when the large lymphatics were located around large vessels such as the SMV, SMA, and aorta, the healing process was not completed when oral feeding was resumed, allowing for a loss of intraabdominal lymph enriched in chylomicrons. Specifically, ELF can result from intraoperative injury to lymphatic vessels located near the origin of major mesenteric vessels which remain incompletely sealed postoperatively. In this context, different lymphatic vessel sealing techniques (monopolar, ultrasound, and radiofrequency) could play a role in ELF development, even though no differences were demonstrated among those energy devices in previous studies. Upon resumption of an oral diet, the lymphatic load increases, and leakage of chyle-rich fluid occurs through these unsealed vessels, manifesting as ELF. Several additional factors are associated with the occurrence of an ELF after colorectal surgery, such as older age [23], preoperative chemotherapy [27], number of harvested lymph nodes [28], number of positive nodes [29], manipulation of the para-aortic area [18], concomitant vascular resection [19], increased intraoperative blood loss [27], and right hemicolectomy [23]. Laparoscopic lymph node dissection during CME was found to be a potential risk factor for the occurrence of a chylous fistula [4]. The role of laparoscopic surgery in increasing the risk of ELF was explained via ultrasonic, monopolar, and bipolar instruments during dissection of the main lymphatic vessels, which, in open surgery, are more often ligated or sutured [30]. In our series of 279 surgically treated colorectal cancer patients, the rate of minimally invasive surgery was 92.1%. Among ELF patients, 86.7% of patients were treated with a minimally invasive approach using ultrasonic, monopolar, and bipolar instruments, especially in the dissection of the main lymph node, at the origin of the vessels, and in preparing a correct and uninterrupted plan for mesocolic/mesorectal excision. Dissection using monopolar diathermy during lymphadenectomy does not seem to perfectly seal or close the lymphatic vessels during excision [5].

In our series, 60% of patients who developed an ELF underwent right colon resection and the rate of right colon cancer in ELF was higher than the rate recorded in no ELF group. Moreover, in the present series of 15 ELF patients, 4 patients (26.7%) were affected by T4 colorectal cancer, slightly higher than the rate of T4 cancer reported in No ELF patients (17.0%). The median number of harvested lymph nodes among the ELFs (23) was significantly greater than the minimum number of nodes (12), which should be removed to ensure that the resection is oncologically correct, but the median number of harvested nodes in no ELFs patients was similar (22). In the series reported by Matsuda et al., the occurrence of a chylous fistula was significantly related to the worst oncologic outcomes [5]. However, the negative effect of an ELF on the oncological prognosis was not clear [8]. The pathological stage of the tumour, particularly the pT stage, and the number of harvested lymph nodes were found to be significantly related to the occurrence of an ELF [8]. The extent of lymph node dissection and, more precisely, the dissection of central nodes (D3), the cornerstone of complete mesocolic excision, and total mesorectal excision, seem to represent an aetiologic factor for the occurrence of ELF in the colorectal cancer series reported in the literature. The role of lymphadenectomy in the etiology of ELFs could be explained by the geographic distribution of the largest series of ELFs in the literature, which are from East Asian centres (Japanese, Korean, and Chinese centres). In these centres, extensive lymphadenectomy (such as D3 lymphadenectomy) has a large diffusion and is commonly practised in these countries [10]. With the introduction of complete mesocolic excision and central vascular ligation in Western countries, the number of ELFs has increased.

An important aspect which we wanted to analyze in our series was the management of the ELF. In the present series, the first therapeutic approach for all patients was a conservative approach based on rapid recovery and TPN alone. In all cases, we observed a fast resolution of the ELF within 24 h after resuming fasting. However, in more than 40% of cases, after restarting the MCT diet, a fistula recurrence occurred. Nevertheless, in such cases, after resuming fasting and TPN, the fistula resolved within 24 h. After 5 days of TPN and fasting, all recurrent fistulas were resolved. No recurrence occurred after restarting the MCT diet. To reduce the risk of ELF recurrence for all patients, we recommend continuing an MCT diet for another 2 weeks after resuming fasting. Due to its low frequency, there are no indications on how to prevent ELF or guidelines for its management; current treatments remain controversial [31]. However, based on evidence in the literature, treatment of the ELF should follow a step-up approach from conservative treatment for several weeks to operative surgical treatment if the conservative approach fails. The rationale of a nonoperative approach is the self-limiting character of the ELF, which can generally heal within 2–3 weeks. The hypothesis is that the patient/s lymphatics can take more time to heal than the conventional time employed to resume oral intake. The self-limiting nature of ELF has also been demonstrated by a success rate after a conservative approach of more than 70% [32,33], with several series that yielded a 100% success rate after conservative management [34,35]. The conservative management of ELF is based on fast resumption (if necessary); parenteral nutrition; the application of drugs such as somatostatin analogues (if necessary); and an MCT diet, rarely, if diffuse chyloperitoneum and paracentesis occur [33]. An MCT diet should represent the first therapeutic step in the management of ELFs. The rationale for the success of an MCT diet is that MTCs directly pass from the intestine into the portal circle, bypassing the lymphatic system. In contrast, long-chain triglycerides must pass from the intestine into the lymphatic system to be completely metabolized [36,37,38]. The success rate of an MCT diet alone in the management of ELFs is approximately 75% [39]. In contrast, the rate of success in the treatment of ELFs with TPN alone seems to vary between 77% and 100% [18,40]. The utility of TPN in the management of ELF is that TPN allows for complete bowel rest with a consequent reduction in lymph production. Moreover, TPN guarantees sufficient supplies of proteins, vitamins, and electrolytes, which preserve protein synthesis and increase peritoneal effusion absorption [35]. Considering TPN’s high rate of success in the management of ELFs, several authors have suggested applying TPN immediately after the clinical diagnosis of an ELF [41,42,43]. Notable reservations regarding the use of TPN in ELF management are due to the risk of the same complications related to TPN, such as infection, thrombosis, cholestasis, and mucosal integrity disorders [32]. Another conservative approach for the management of ELFs is associated therapy with somatostatin analogues such as octreotide (most frequently used in clinical practice due to having a longer half-life than somatostatin), an MCT diet, or TPN, especially for high-output or refractory ELFs [31,44]. The success rate of somatostatin analogues varies from 85% to 100%, with a significant decrease in ELF output within 24–72 h after the start of octreotide therapy [31,41,45]. In refractory ELF, a more invasive approach is needed but not always effective. Bipedal lymphangiography with lipiodol has an occlusion rate between 35% and 70%, which is greater for low-output (<500 mL per day) ELFs [46,47,48]. Surgery is the main treatment for refractory ELF and should be considered when leakage is sustained for more than 2 weeks, when the output is more than 1 litre for more than 1 week, and when the patient starts to experience metabolic complications [49]. Surgical management includes unassisted surgery with or without the use of fibrin glue [50,51,52,53,54,55]. Other surgical procedures described for the management of ELFs include peritoneovenous shunts and transabdominal catheterization of the cisterna chyli. However, the effectiveness of these procedures remains unclear due to the small sample size of these studies [19,40].

## 5. Limitations of the Study

This study has several limitations, primarily related to the relatively rare incidence of this complication, especially in Western countries. The main limitation is the sample size (15 cases), which, although observed among 30 months and 279 colorectal resections, does not allow for statistical analysis of risk factors associated with the development of ELF. A related limitation is the inability to perform a statistically meaningful comparison between the 15 patients who developed ELF and the 264 patients who did not. Additionally, the retrospective nature of the study further limits the strength of our evaluation. Nevertheless, despite these limitations, this study currently represents the largest collection of ELF cases reported in the literature within a Western setting and may serve as a basis for future multicenter studies aimed at effectively identifying predictive factors for ELF and standardizing its therapeutic management.

## 6. Conclusions

Improving the oncological results in colorectal cancer currently requires the increasingly extensive application of CME, TME, and central lymph node dissection, exposing patients to a greater risk of developing complications such as ELF, as reported in Eastern countries. This result suggests that chylous fistulas are more likely to be radical when they occur in greater numbers. Conservative treatment based on rapid recovery and the use of a TPN and MCT diet appears to represent a valid and effective alternative to more invasive surgical treatment, which could be necessary for the management of refractory or high-output ELFs. However, the necessity of fast surgery and TPN seems to negatively influence the length of postoperative stay.

## Figures and Tables

**Table 1:** Baseline characteristics of surgically treated colorectal cancer patients.

Variables	Study Population (N: 279)
Age (year, median (IQR))	73 (65–81)
Sex (M:F)	154:125
BMI (kg/m^2^, median (IQR))	24.80 (21.98–27.68)
Charlson Comorbidity Index (median (IQR))	6 (4–7)
ASA score > 2, n (%)	93 (33.3)
Tumour site, n (%)	
Right colon cancer	81 (29.0)
Hepatic flexure colon cancer	27 (9.7)
Transverse colon cancer	11 (3.9)
Splenic flexure colon cancer	10 (3.6)
Left colon cancer	76 (27.3)
Intraperitoneal rectal cancer	24 (8.6)
Extraperitoneal rectal cancer	50 (17.9)
Preoperative neoadjuvant chemoradiotherapy, n (%)	48 (17.2)
Minimally invasive approach, n (%)	257 (92.1)
Conversion rate, n (%)	12 (4.3)
Tumour diameter, cm (median (IQR))	3 (2–4.5)
pT4 rate, n (%)	48 (17.2)
Harvested lymph nodes (median (IQR))	22 (16–30)
Positive nodes, n (%)	99 (35.4)
Diet resuming after surgery (days, median (IQR))	2 (2–2)
Short-term overall postoperative morbidity, n (%)	97 (34.7)
Clavien–Dindo Grade ≥ 3, n (%)	20 (7.1)
Anastomotic leak, n (%)	8 (2.8)
Postoperative length of stay (days, median (IQR))	5 (5–7)
Reoperation rate within 30 days, n (%)	15 (5.3)
Short-term postoperative mortality, n (%)	0 (0)

**Table 2:** Baseline characteristics and perioperative features of patients who developed an ELF (ELF group) and patients who did not develop an ELF (No ELF group).

**Variables**	**ELF Group (15)**	**No ELF Group (264)**
Age (year, median (IQR))	76 (65–80)	73 (65–81)
Sex (M:F)	9:6	145:119
BMI (kg/m^2^, median (IQR))	25.47 (23.00–28.32)	24.78 (21.97–27.68)
Charlson Comorbidity Index (median (IQR))	6 (5–8)	6 (4–7)
ASA score > 2, n (%)	5 (33.3)	89 (33.7)
Tumour site, n (%)		
Right colon cancer	9 (60)	100 (37.9)
Transverse colon cancer	1 (6.7)	17 (6.4)
Left colon cancer	4 (26.6)	83 (31.4)
Intraperitoneal rectal cancer	-	14 (5.4)
Extraperitoneal rectal cancer	1 (6.7)	50 (18.9)
Preoperative neoadjuvant chemoradiotherapy, n (%)	1 (6.7)	47 (17.8)
Mini-invasive approach, n (%)	14 (93.3)	243 (92.0)
Conversion rate, n (%)	1 (6.6)	11 (4.16)
Tumour diameter (cm, median (IQR))	3 (2.15–4.5)	3 (2–3.9)
pT4 rate, n (%)	4 (26.7)	45 (17.0)
Harvested lymph nodes (n., median (IQR))	23 (21–29)	22 (16–30)
N. of patients with positive nodes, n (%)	7 (46.7)	92 (34.9)
Diet resuming after surgery (median (IQR))	2 (1–2)	2 (1–2)
Short-term overall postoperative morbidity		
Clavien–Dindo Grade ≥ 3, n (%)	0	16 (6.1)
Anastomotic Leak, n (%)	0	8 (3.0)
Postoperative length of stay (days, median (IQR))	11 (10–14)	5 (5–6)
Reoperation rate within 30 days, n (%)	0	15 (5.7)
Short-term postoperative mortality, n (%)	0	2 (0.7)

## Data Availability

Data are unavailable due to privacy restrictions.
